# Genomic events stratifying prognosis of early gastric cancer

**DOI:** 10.1007/s10120-024-01536-z

**Published:** 2024-07-19

**Authors:** Chiara Molinari, Leonardo Solaini, Francesca Rebuzzi, Gianluca Tedaldi, Davide Angeli, Elisabetta Petracci, Dusan Prascevic, Jan Ewald, Erhard Rahm, Matteo Canale, Martinelli Giovanni, Anna Tomezzoli, Maria Bencivenga, Maria Raffaella Ambrosio, Daniele Marrelli, Paolo Morgagni, Giorgio Ercolani, Paola Ulivi, Luca Saragoni

**Affiliations:** 1 Biosciences Laboratory, IRCCS Istituto Romagnolo Per Lo Studio Dei Tumori (IRST) “Dino Amadori”, Meldola, FC Italy; 2 Department of Medical and Surgical Sciences, University of Bologna, Bologna, Italy; 3 General and Oncologic Surgery, Morgagni-Pierantoni Hospital, AUSL Romagna, Forlì, Italy; 4 Biostatistics and Clinical Trials Unit, IRCCS Istituto Romagnolo Per Lo Studio Dei Tumori (IRST), “Dino Amadori”, Meldola, FC Italy; 5 Center for Scalable Data Analytics and Artificial Intelligence (ScaDS.AI), Dresden/Leipzig University, Humboldtstr. 25, 04105 Leipzig, Germany; 6 Department of Hematology and Sciences Oncology, Institute of Haematology “L. and A. Seràgnoli”, S. Orsola University Hospital, Bologna, Italy; 7 Department of Pathology, University of Verona, Verona, Italy; 8 Unit of Upper GI Surgery, University of Verona, Verona, Italy; 9 Pathology Unit, University of Siena, Siena, Italy; 10 Surgery Unit, University of Siena, Siena, Italy; 11 Pathology Unit, Morgagni-Pierantoni Hospital, Forlì, Italy

**Keywords:** EGC, Pen, *ARID1A*, *LRP1B*, Prognosis

## Abstract

**Background:**

The purpose of the study was to conduct a comprehensive genomic characterization of gene alterations, microsatellite instability (MSI), and tumor mutational burden (TMB) in submucosal-penetrating (Pen) early gastric cancers (EGCs) with varying prognoses.

**Methods:**

Samples from EGC patients undergoing surgery and with 10-year follow-up data available were collected. Tissue genomic alterations were characterized using Trusight Oncology panel (TSO500). Pathway instability (PI) scores for a selection of 218 GC-related pathways were calculated both for the present case series and EGCs from the TCGA cohort.

**Results:**

Higher age and tumor location in the upper-middle tract are significantly associated with an increased hazard of relapse or death from any cause (*p* = 0.006 and *p* = 0.032). Even if not reaching a statistical significance, Pen A tumors more frequently present higher TMB values, higher frequency of MSI-subtypes and an overall increase in PI scores, along with an enrichment in immune pathways. *ARID1A* gene was observed to be significantly more frequently mutated in Pen A tumors (*p* = 0.006), as well as in patients with high TMB (*p* = 0.027). Tumors harboring *LRP1B* alterations seem to have a higher hazard of relapse or death from any cause (*p* = 0.089), being mutated mainly in relapsed patients (*p* = 0.093).

**Conclusions:**

We found that the most aggressive subtype Pen A is characterized by a higher frequency of *ARID1A* mutations and a higher genetic instability, while *LRP1B* alterations seem to be related to a lower disease-free survival. Further investigations are needed to provide a rationale for the use of these markers to stratify prognosis in EGC patients.

**Supplementary Information:**

The online version contains supplementary material available at 10.1007/s10120-024-01536-z.

## Introduction

Gastric cancer (GC) incidence has decreased in Western countries, but this neoplasm is still the fifth solid cancer for frequency and the fourth leading cause of cancer-related death worldwide [1]. Early gastric cancer (EGC), classically defined by the Japanese Cancer Association as a carcinoma limited to the mucosa and/or submucosa regardless of the lymph node status [2], is generally associated with a favorable outcome, with 10-year survival of around 90% [3, 4]. In Europe, the incidence of EGC is much lower than in Asian countries and it represents 10–15% of all GC [5–7].

Despite the generally good prognosis, a subgroup of EGC seems to be associated with poor prognosis. In particular, a multicenter GIRCG (Italian Gastric Cancer Research Group) study has demonstrated that the Kodama’s classification could identify a subgroup of EGC patients who have a significantly poorer prognosis according to the type of submucosal invasion [3, 8]. Submucosa-penetrating tumor (Pen) A type, reported to represent around 20% of all EGCs, was, in fact, significantly associated with lower 10-year survival (78.2%) when compared with other submucosal non-Pen A (91.5%) and mucosal (94.2%) EGC. It was also found that Pen A type differed from other EGCs for various clinico-pathological features. As such, the proportion of patients with  > 60 years, with tumor size  > 2 cm and pN+  status was found to be higher in Pen A types [3]. According to these features and giving its high lymphatic spread, Pen A type EGCs should always be approached with radical gastrectomy plus D2 lymphadenectomy to guarantee the most appropriate oncologic treatment. However, to date, it is difficult to detect a Pen A type before surgical or endoscopic treatment as the type of submucosal involvement could be defined only after resection by the pathologist.

Recent studies tried to characterize EGC at the molecular level, analyzing their expression profiles or genetic alterations, but considering mainly intramucosal tumors, together with premalignant lesions to dissect mechanisms underlying GC cancerogenesis [9–11]. Some other studies aimed to find biomarkers of aggressiveness, either focusing on the EGC tumoral component and on the tumor microenvironment [12–15], but to date the relative contributions of them in the prognostic stratification of EGCs patients warrant further investigation.

Within the same aim, to our knowledge, a wide genomic characterization of gene alterations, microsatellite instability (MSI) status and tumor mutational burden (TMB) of submucosal-penetrating EGCs at different prognosis is still missing and this was the main purpose of our study.

## Methods

### Study design

This was an observational study conducted at the Istituto Romagnolo per lo Studio dei Tumori “Dino Amadori” on cases with a histological diagnosis of penetrating EGC and surgically treated from 1990 to 2014 by three centers: Morgagni-Pierantoni Hospital of Forlì, University of Verona and University of Siena (Italy). All patients underwent surgery with D2 lymph node dissection. The analyses were performed on patients with adequate tumoral tissue samples and clinico-pathological and follow-up data. Tumors were classified according to Kodama classification [8] in submucosal-penetrating Pen A, referring to EGCs massively invading the submucosa with a nodular pattern and measuring less than 4 cm, and submucosal-penetrating Pen B, which includes EGCs invading the submucosa with a sawtooth pattern and measuring less than 4 cm. The study was conducted in accordance with the Declaration of Helsinki and all participants provided written informed consent.

### DNA extraction

The genomic DNA (gDNA) extraction was performed using QIAamp DNA FFPE Tissue Kit (Qiagen, Hilden, Germany) starting from macrodissected formalin-fixed paraffin-embedded (FFPE) slices containing at least 50% of tumoral cells. Manufacturer recommendations were followed for quantitative and qualitative evaluation of DNA quality. The gDNA was quantified with Qubit® dsDNA BR Assay on Qubit 3.0 fluorometer (Invitrogen, Carlsbad, CA) and diluted for the subsequent molecular analyses.

### Next-generation sequencing (NGS) analysis

All gDNA samples were characterized for a wide variety of genetic alterations, including single nucleotide variants (SNVs) of 523 genes and copy number variants (CNVs) of 49 gene, as well as MSI and TMB, using Trusight Oncology 500 DNA kit (Illumina) (Supplementary Table 1). Forty ng of gDNA was fragmented using the ME220 ultrasonicator (Covaris). The libraries were prepared and enriched for the 523 genes targeted by the panel following the manufacturer’s instructions. In each run, eight final libraries were pooled and loaded on NextSeq^™^ 550 sequencer (Illumina). Data were analyzed using the Illumina TSO500 Local app software version 2.0.1.4 (filters: median insert size ≥ 75; percentage of target bases with coverage greater than 100X ≥ 75%). Variants were annotated using ANNOVAR (version 2020-06-08) [16] (filters: i) read depth ≥ 100X; ii) variant allele frequency  ≥ 5%; iii) exclusion of non exonic, non splicing and synonymous variants) and Varsome tool (version 11.7.2) [17] to classify them according to ACMG/AMP guidelines [18].

The selected cutoff values were  ≥ 10 mut/Mb to define high TMB and  > 20% of unstable microsatellites sites to define MSI [19, 20]. The minimum number of usable microsatellite sites to define MSI status was 40. The OncoPrint and Fig. 5a were built with ComplexHeatmap R package [21].

### Pathway instability analysis

SNV and CNV data were used to calculate pathway instability (PI) scores for a selection of 218 GC-related pathways in order to estimate their mutagenic disruptions [22] (Supplementary material). The GC-related pathways were selected based on the Reactome pathways enrichment analysis using the list of altered genes across EGC patients [23], using the Reactome Pathway analysis tool [24]. Alterations classified as benign were excluded.

The resultant high-dimensional matrix of PI scores was reduced to two dimensions, using the t-distributed stochastic embedding (t-SNE) dimensionality reduction algorithm [25]. This representation of the PI scores was clustered using the density-based spatial clustering (DBSCAN) method [26]. Both algorithms were applied via the scikit-learn 1.1.3 package [27]*.* The implementation of the pathway instability analysis can be found at 10.5281/zenodo.10816958.

## Statistical analysis

Participant characteristics were summarized by means of descriptive statistics such as mean ± standard deviation (SD) or median, first (IQ) and third (IIIQ) quartile, as well as minimum and maximum values for continuous variables, and frequencies and percentages for categorical ones. Student’s *t* test or the Mann–Whitney *U* test and the Chi-square or the Fisher’s exact test, as appropriate, were used to compare baseline characteristics between the Pen A and Pen B groups. The Cox proportional hazard (PH) model was used to investigate the association between baseline covariates and disease-free survival (DFS). The proportional hazards assumption was tested using Schoenfeld residuals. Results were reported as hazard ratios (HRs) and 95% confidence intervals (CIs). DFS was defined as the time since surgery to disease recurrence and death from any cause, whichever occurred first.

For identifying the defining pathways of obtained clusters, the Wilcoxon rank-sum test was used. *P* values were corrected for multiple testing using the Benjamini/Hochberg procedure, using a cutoff of 0.05 for establishing significance. To test whether unsupervised clustering captures important clinical features, Fisher’s exact test was used to compare the distribution of clinical parameters between the clusters. Lastly, the distribution of PI scores was directly compared across various clinical groupings (TMB class, MSI class, Kodama class and relapse status) using the Wilcoxon rank-sum test.

The analyses were carried out using STATA 15.1 (college station, Texas, USA), R version 4.1.0 statistical software (http://cran.r-project.org/) and Python version 3.8.16 (Python Software Foundation, https://www.python.org/).

## Results

### Clinico-pathological characteristics and prognosis of EGC patients

**Fig. 1:**
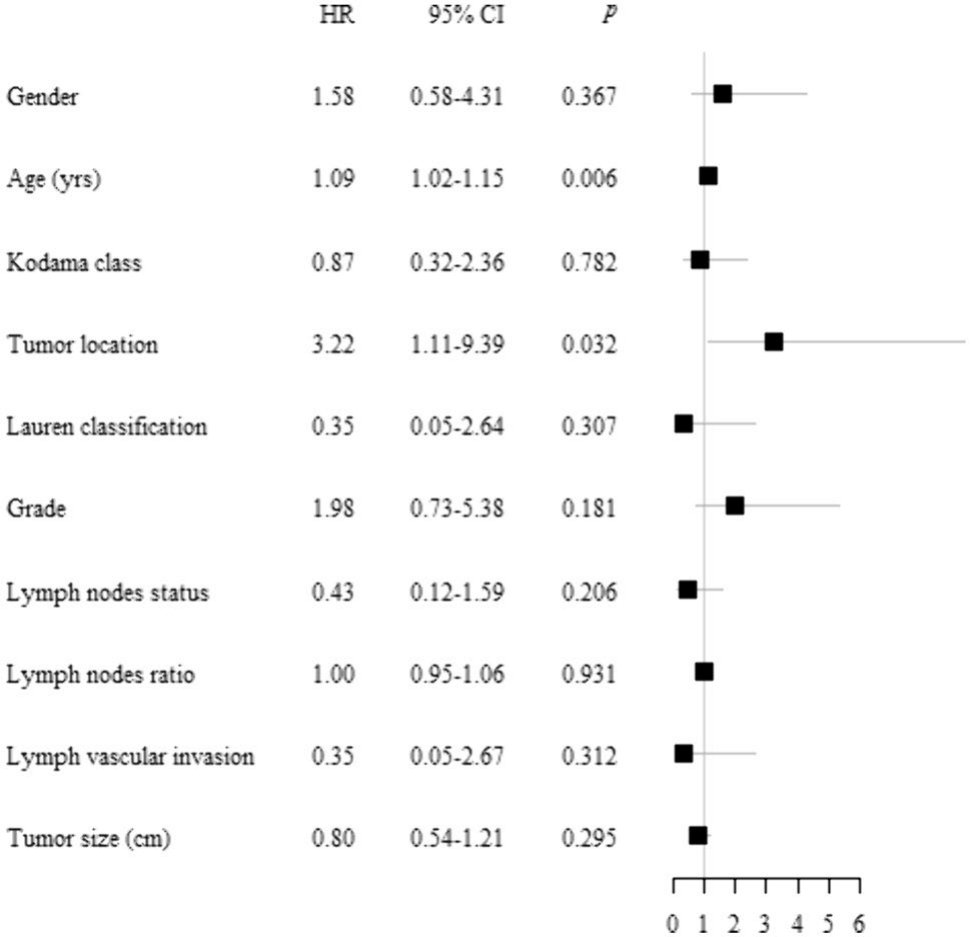
Forest plot. Univariate Cox regression analysis for disease-free survival. The following reference categories were used: “female” for gender; “Pen B” for Kodama classification; “antrum” for tumor location; “intestinal” for Lauren classification; “G1+G2” for grade; “N0” for lymph node status; “Absent” for lymphovascular invasion. Age, Tumor size, and lymph node ratio are reported as continuous variables. *HR* hazard ratio, *CI* confidence interval, *p* *p*-value

A total of 27 patients were considered in the analyses. Most were females, with a mean age at EGC diagnosis of 67 $\pm$ 11.7 years. Tumors were mainly classified as intestinal type and did not involve lymph nodes. Among patients, 14 were diagnosed with Pen B (52%) and 13 (48%) with Pen A tumors, according to the Kodama’s classification; 9 (33%) and 7 (26%) patients had a recurrence or death as first event, whereas 11 (67%) patients were disease free/alive within 10 years of follow-up. No statistically significant differences were observed between Pen B and Pen A tumors (Table 1). At univariate analyses using Cox PH model, higher age and tumor location in the upper-middle tract were associated with an increased hazard of relapse or death from any cause (*p* = 0.006 and *p* = 0.032, respectively), as shown in the forest plot (Fig. 1). Moreover, patients with higher tumor grade seem to have about a double risk of relapsing or dying, even if the small case series does not permit statistical significance.

**Table 1:** Clinico-pathological characteristics of patients by infiltration subtypes

Characteristics	Pen B (*n* = 14) (%)	Pen A (*n* = 13) (%)	Total (*n* = 27) (%)	*p*-value
Age at diagnosis (yrs)				
Mean ± sd	65.7 ± 11.0	68.3 ± 12.8	67.0 ± 11.7	0.481
Min–max	45–84	41–87	41–87	
Gender				
F	7 (50.0)	8 (61.5)	15 (55.6)	0.547
M	7 (50.0)	5 (38.5)	12 (44.4)	
Tumor location				
Upper-middle^a^	8 (57.1)	5 (38.5)	13 (48.2)	0.332
Lower^b^	6 (42.9)	8 (61.5)	14 (51.8)	
Macroscopic classification				
Mixed	1 (7.1)	0	1 (3.7)	0.232
I	2 (14.3)	2 (15.4)	4 (14.8)	
IIa	3 (21.4)	0	3 (11.1)	
IIc	7 (50.0)	7 (53.9)	14 (51.9)	
III	1 (7.1)	4 (30.8)	5 (18.5)	
Lauren classification				
Intestinal	11 (78.6)	12 (92.3)	23 (85.2)	1.000
Diffuse or mixed	3 (21.4)	1 (7.7)	4 (14.8)	
Grade				
G1 + G2	8 (61.5)	6 (46.2)	14 (53.9)	0.431
G3	5 (38.6)	7 (53.8)	12 (46.1)	
Unknown/missing	1		1	
pN status				
pN0	11 (78.6)	8 (61.5)	19 (70.4)	0.420
pN +	3 (21.4)	5 (38.5)	8 (29.6)	
Lymph nodes ratio^#^				
Median [IQ–IIIQ]*	0 [0–0]	0 [0–7.1]	0 [0–4]	0.254
Min–max	0–23.5	0–45.5	0–45.5	
Lymphovascular invasion				
Absent	11 (78.6)	12 (92.3)	23 (85.2)	0.596
Present	3 (21.4)	1 (7.7)	4 (14.8)	
Tumor size (cm)				
Median [IQ–IIIQ]*	2 [1.7–3.5]	2 [1.8–3.5]	2 [1.7–3.5]	0.574
Min–max	1–3.5	1–6	1–6	

*IQ: first quartile; IIIQ: third quartile

^#^Lymph node ratio defined as the number of involved nodes divided by the number of lymph nodes examined

^a^Upper-middle: fundus and body

^b^Lower: ANTRUM

### Molecular profiling of EGCs

**Fig. 2:**
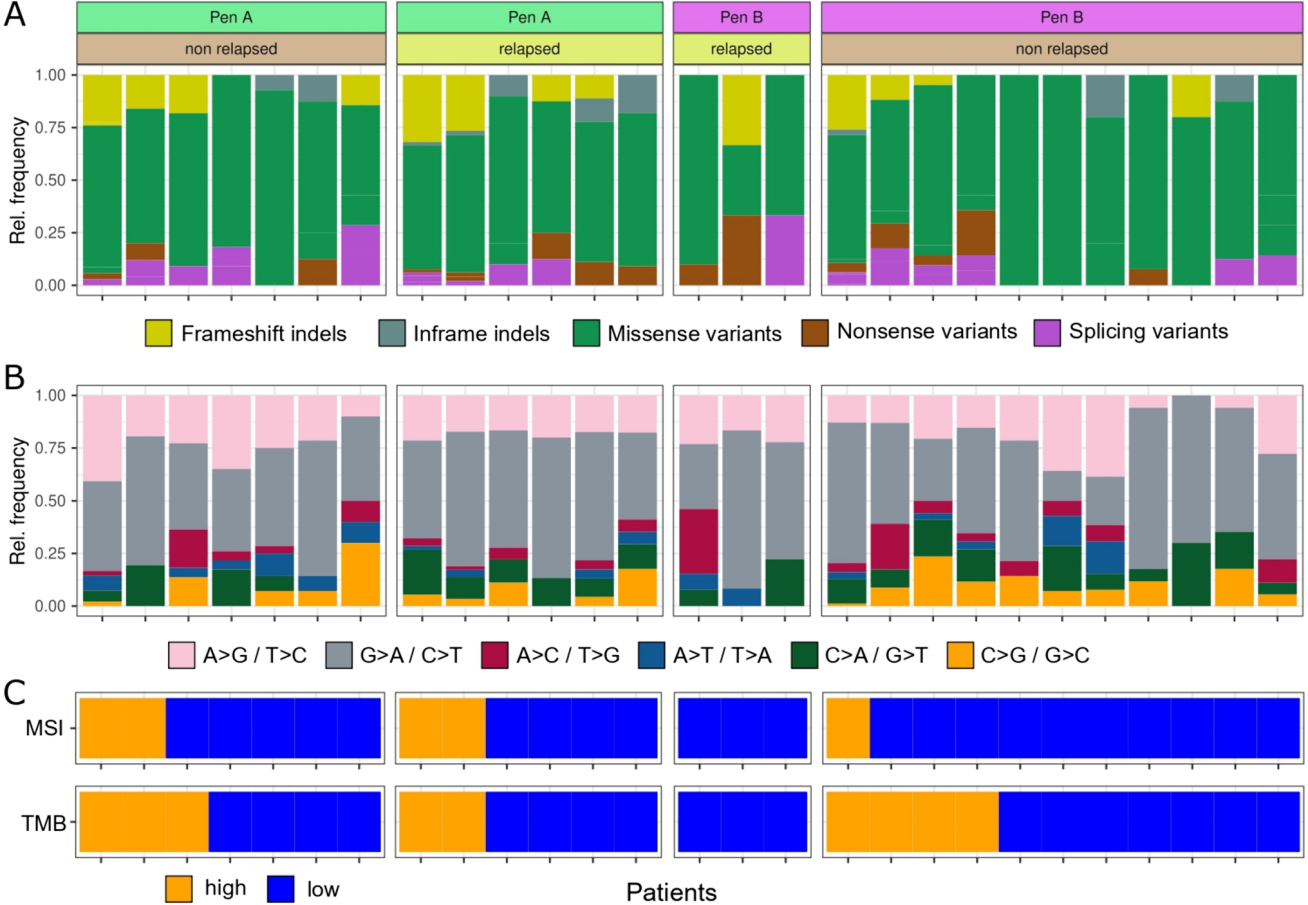
Mutational characterization of EGCs based on DNA sequencing. **a** For each patient (x-axis, ordered by TMB) relative frequency of variant types is shown for mutations in exonic regions of genes. **b** Relative frequency of SNV types. **c** MSI and TMB status  of EGC patients. Patients are grouped by Kodama classification and relapse status in all subfigures

NGS analysis revealed a total of 904 variants. Among them, 200 variants (22.1%) were predicted as pathogenic/likely pathogenic, 372 (41.2%) as variants of uncertain significance (VUS), and 332 (36.7%) as benign/likely benign. Regarding the effect on the protein, 103 variants (11.4%) were frameshift deletions/insertions, 17 (1.9%) in-frame deletions/insertions, 27 (3.0%) nonsense mutations, 721 (79.8%) missense mutations, and 36 (4%) splice site mutations (Fig. 2a). Regarding mutation type, 73.4% were transitions and 26.6% were transversions (Fig. 2b). No significant differences between Pen A and Pen B tumors as well as relapsed and non-relapsed status were observed.

TMB and MSI were significantly associated with 100% of MSI high (> 20%) patients showing also a high TMB (≥ 10), Fisher’s exact *p* = 0.002. Although no statistically significant difference was observed, Pen A presented higher median TMB values compared to Pen B (8 [IQ-IIIQ 4.8 – 25.3] vs 5.2 [IQ-IIIQ 2.4 – 11.1] (*p* = 0.120)), as well as a higher number of MSI subtype tumor, defined according to the selected cut off (*p* = 0.165) (Fig. 2c and Supplementary Table S2).

**Fig. 3:**
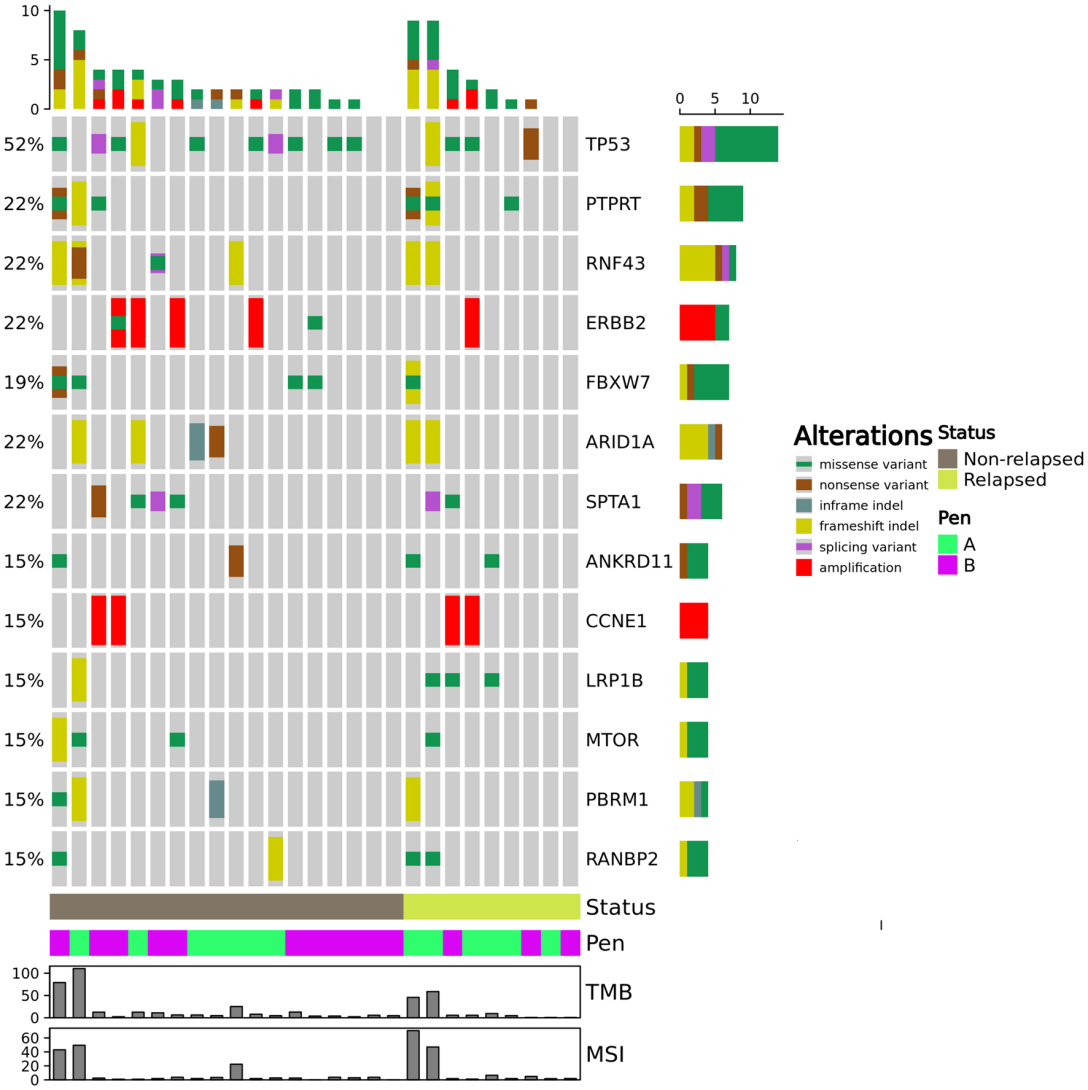
The OncoPrint chart.  The heatmap represents genomic alterations including pathogenic variants and VUS, found in at least four patients. Patients are ordered by non-relapsed or relapsed status. The chart was built by ComplexHeatmap R package

**Fig. 4:**
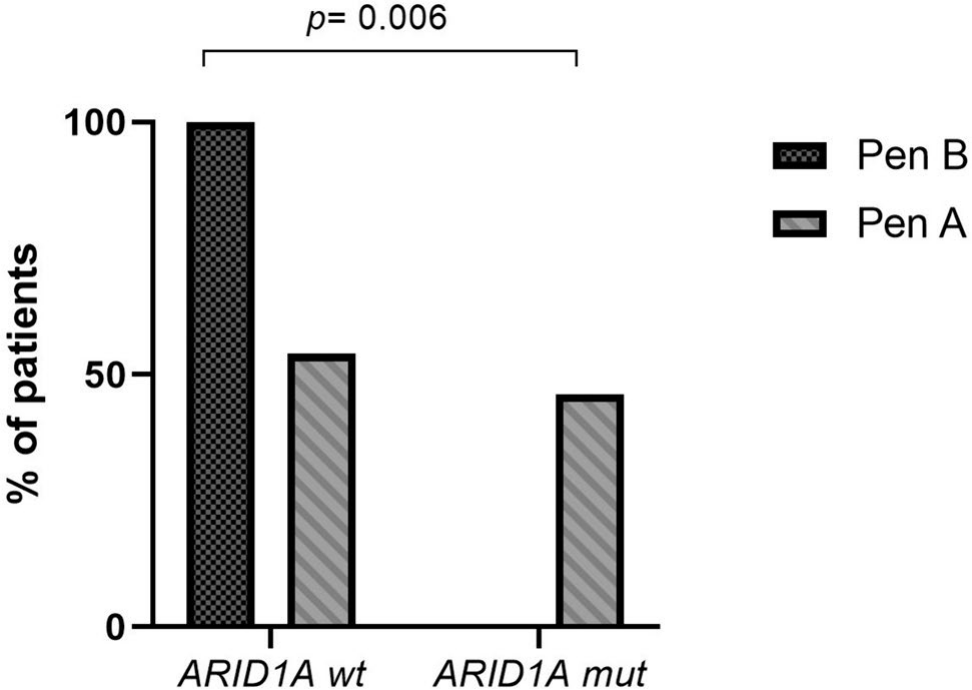
*ARID1A* status in EGC patients. The histogram represents the percentage of *ARID1A* alterations in the two different subtypes of Pen tumors. *wt* wild-type, *mut* mutant

Genomic analysis revealed that the most frequently mutated genes were *TP53*, protein tyrosine phosphatase receptor type T (*PTPRT*), E3 ubiquitin ligase ring finger protein 43 (*RNF43*), F-box and WD repeat domain-containing7 (*FBXW7*)*,* AT-rich interaction domain 1A (*ARID1A)* and spectrin alpha, erythrocytic 1 (*SPTA1*)*,* as shown in Fig. 3. Twenty-two percent of patients presented erb-b2 receptor tyrosine kinase 2 (*ERBB2)* gene alterations, 5/27 (18%) being *ERBB2* amplified. Similarly, cyclin E1 (*CCN1E*) was amplified in 15% of tumors. Of interest, mutations in *ARID1A* were found only in Pen A tumors (*p* = 0.006), 4/6 of them being frameshift substitutions classified as “likely pathogenic”, the remaining 2 classified as variants of uncertain significance (Table 2 and Fig. 4). A significant association between *ARID1A* alterations and high TMB (*p* = 0.027) and microsatellite instability (*p* = 0.056) has been observed. No other statistically significant molecular differences between Pen A and Pen B were observed.

**Table 2:** *ARID1A* mutations

Pts ID	*ARID1A* mutations^1^	Exon	Domain	Significance^2^	Consequence	Tumor type	TMB status	MSI status
EGC11	G277R	1	Not functional domain	Likely pathogenic	Frameshift substitution	Pen A	TMB-High	MSI
EGC12	Q1327A	16	Not functional domain	Likely pathogenic	Frameshift substitution	Pen A	TMB-High	MSI
EGC19	P1326R	16	Not functional domain	Likely pathogenic	Frameshift substitution	Pen A	TMB-High	MSI
EGC7	P1451R	18	HIC1 domain	Likely pathogenic	Frameshift substitution	Pen A	TMB-High	MSS
EGC1	E2036del	20	GR-binding domain	Uncertain significance	Nonframeshift substitution	Pen A	TMB-Low	MSS
EGC10	W2091X	20	GR-binding domain	Uncertain significance	Stopgain	Pen A	TMB-Low	MSS

^1^ Refer to isoform NM_006015. ^2^ ACMG classification

In patients with a high tumor mutational burden, other significantly mutated genes were *APC* (*p* = 0.029), and *ARID2* (*p* = 0.029). A significant association was also observed between MSI and *ANKRD11* (*p* = 0.013) and *RNF43* mutations (*p* < 0.0001).

The broad molecular characterization of the EGC series did not reveal any other significant associations with clinico-pathological characteristics, except for *TP53* mutations which were more frequently found in intestinal-type EGC and in males (*p* = 0.041 and *p* = 0.054) and for the association between the low-density lipoprotein receptor-related protein 1b (*LRP1B*) mutations and higher age (*p* = 0.020). Despite not reaching statistical significance, tumors harboring *LRP1B* alterations seem to have a higher hazard of relapse or death from any cause (HR = 2.73 95% CI: 0.857–8.703 *p* = 0.089), being mutated mainly in relapsed patients (*p* = 0.093).

### Pathway analysis of EGC

**Fig. 5:**
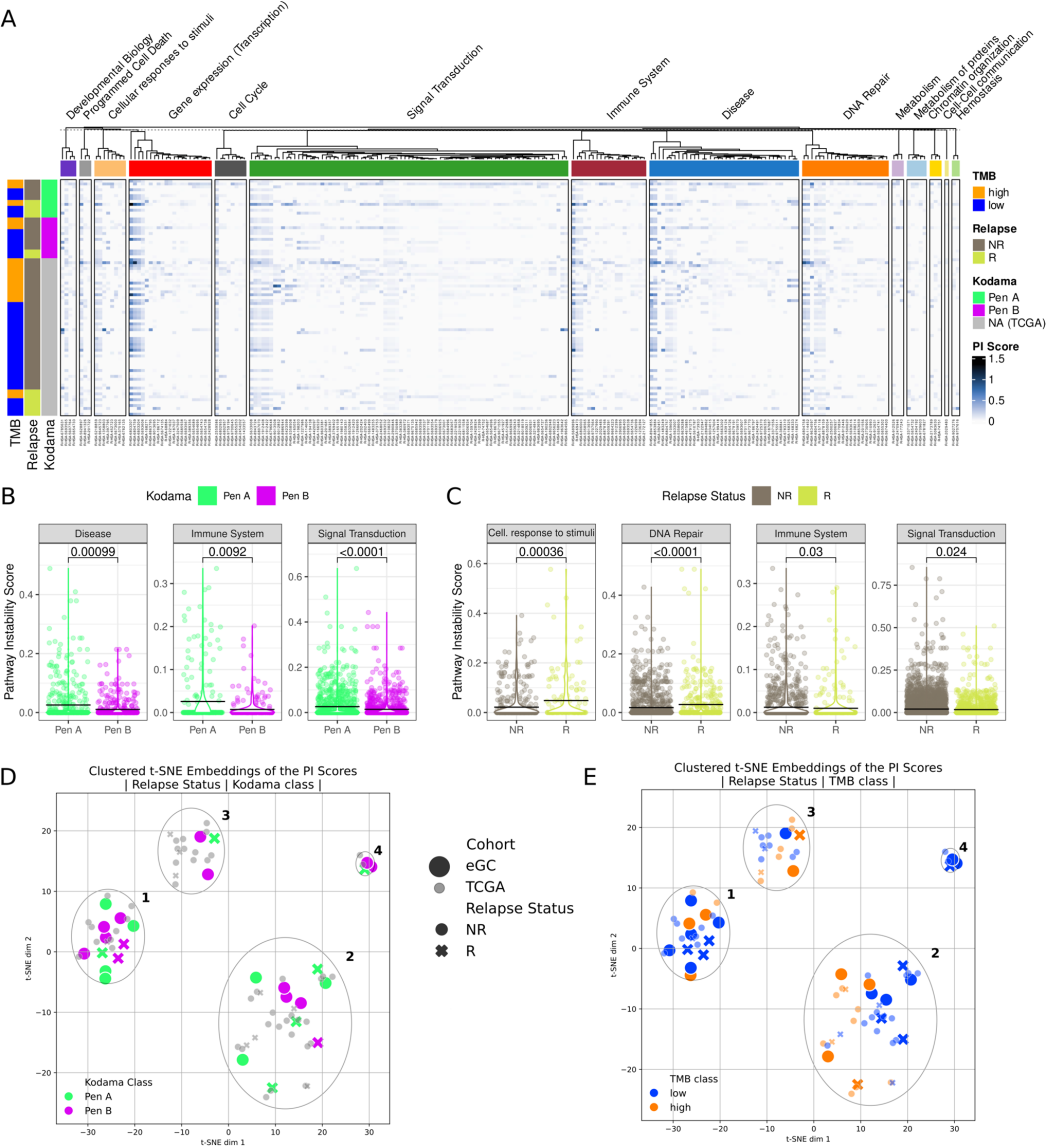
Pathway instability analysis. **a** Heatmap representation of the 218 GC pathways identified with the Reactome enrichment analysis. Pathways are sorted in columns and grouped according to their corresponding Top Level pathways, with hierarchical clustering applied within the groupings. TMB and MSI class, as well as relapse status and Kodama classification are annotated on the left side for all patients. **b**, **c** PI score distribution across  **b** Kodama classification subtypes and **c** relapse status, shown for pathways grouped according to selected top level pathways. Each data point indicates a PI score value for an individual GC-related pathway, for each patient separately. Violin plots with horizontal black lines marking the median values convey the overall distribution of PI scores between different patient groupings. Note that since no information on Kodama classification was available for EGC-TCGA patients, these patients are excluded from comparisons between Pen A and Pen B groups. **d**, **e** Two-dimensional t-SNE representation of PI scores obtained from 218 pathways of interest, for the combined case series of EGC and TCGA EGC. Clustering overlaid with patients’ **d** Kodama classification and **e** TMB classification

The enrichment analysis revealed no statistically significant associations after correction for multiple testing between a pathway’s PI score and Kodama classification at the detailed level with 218 GC-related pathways. The results of the statistical comparison of individual pathway PI scores across different subgroups of patients (TMB and MSI class, Kodama classification, relapse status) are presented in Supplementary Table S3 (available in our Zenodo repository). Visual representation of the PI score distribution for all 218 pathways is presented in Fig. 5a. Individual pathways can be grouped according to their corresponding Top Level Pathway in the Reactome database hierarchy, representing overarching biological processes critical for the functioning of organisms. Such grouping allows the identification of general patterns in how increased pathway instability potentially disrupts these biological functions for different subgroups of patients. Upon grouping the pathways according to their Top Level Pathways, it becomes apparent that higher PI score in pathways belonging to “Disease” (*p* < 0.001), “Immune System” (*p* < 0.001) and “Signal Transduction” (*p* < 0.001) are associated with Pen A classification (Fig. 5b). Association between overall increased PI score and Pen A classification remained significant when considering all pathways together.

To explore in more depth the molecular landscape of early gastric cancer using a bigger case series, the publicly available dataset of Stomach Adenocarcinomas (STAD) from TCGA––PanCancer Atlas was retrieved using the cBioPortal (https://www.cbioportal.org/) [28] and was added to the analysis. To match EGC, only stage I gastric cancer patients with survival data available were included (*n* = 54). The clinico-pathological characteristics of the two cohorts were largely comparable. Notably, the EGC cohort of the present study had a higher percentage of female patients, tumors predominantly located in the lower tract and a greater proportion of pN+ tumors, compared to the TCGA cohort (Supplementary Table S4). Moreover, for the patients belonging to the TCGA cohort, the Kodama classification of the tumor was not available.

PI scores were calculated for the GC-related pathways for a combined cohort of EGC and EGC–TCGA patients (*n* = 81), but no statistically significant association between a pathway’s PI score and the relapse status was observed for any single pathway after correction for multiple testing. However, top level pathways revealed that relapsed patients had significantly higher PI scores in pathways belonging to “DNA Repair” (*p* < 0.001) and “Cellular responses to stimuli” (*p* < 0.001), whereas they had significantly lowered PI scores for “Immune System” (*p* = 0.03) and “Signal Transduction” (*p* = 0.024) (Fig. 5c). Interestingly, whereas disruptions to the “Immune System” and “Signal Transduction” Top Level pathways are significantly enriched in Pen A patients, the exact opposite is true for relapsed patients. However, it is important to note that relapse status was known for all EGC-TCGA patients, whereas Kodama classification was not available, which is why EGC-TCGA patients are absent from comparisons involving Pen status. Furthermore, as we do not observe the expected association between Kodama classification and relapse status, this should not come off as a surprise.

Moreover, the analysis of the combined cohort permits the identification of four main clusters of patients (Fig. 5d, e). Clusters 1–3 are characterized by a majority of TMB low patients, whereas the smallest Cluster 4 is made up of exclusively TMB low patients (*p* = 0.16). Clusters 3 and 4 contain only three patients each with available information on Kodama status, and both feature the same split (2 Pen B vs 1 Pen A). Cluster 1 is characterized by a slight majority (6/11) of Pen B patients, whereas the reverse is true for Cluster 2 with 6/10 patients being Pen A (*p* = 0.44). There were no statistically significant differences among the clusters in the distribution of the main clinico-pathological parameters or disease-free survival (*p* = 0.607) (Supplementary Figure S1).

A closer look showed that the clusters were characterized by specific signatures of disrupted pathways. For example, Cluster 4 is differentiated by disruptions in the pathways belonging mainly to signal transduction by tyrosine kinases. Clusters 2 and 3 share some significantly disrupted pathways such as those involved in transcription, intracellular signaling, metabolism of proteins, DNA repair and programmed cell death. The remaining Cluster 1 had no specific significantly disrupted pathways (Supplementary Table S5- available at 10.5281/zenodo.10816958).

Looking at which genes were most frequently mutated for each cluster provides further credence that PI score-based clustering stratifies patients into groups with distinct molecular characteristics. All patients belonging to Cluster 1 have mutations in *TP53* (100%). After *TP53*, most often mutated genes are *SPTA1* (22.8%), *LRP1B* (18.2%) and *ARID1A* (18.2%), roughly following the overall trend of mutated genes for the cohort. The most frequently mutated gene in Cluster 2 is *ARID1A* (44.44%), which is the 2nd most commonly mutated gene across all clusters with an overall mutation frequency of about 30%. Significant associations for patients with mutated *ARID1A* and TMB and MSI status were observed in the combined case series, but no significant association has been found with the relapse status (*p* = 0.7721). Interestingly, no patients from Cluster 2 have mutated *TP53*. As for Cluster 1, Cluster 3 is *TP53* mutated in all 18 patients (100%), followed by *LRP1B* (33.33%) and *EPHA5* (33.33%). *ARID1A* (22.22%) and *SPTA1* (11.11%), otherwise among the most commonly mutated genes, are notably less mutated in Cluster 3. Lastly, Cluster 4 features a unique mutation signature with the most commonly mutated gene being *RHOA* (100%), followed by *TP53* (20%) and *RNF43* (20%) (Supplementary Table S6).

## Discussion

In the minimally invasive surgery era, EGC may be more easily treated with D1+ lymphadenectomy or with more conservative endoscopic-assisted hybrid procedures. However, a variable outcome has been observed for patients with submucosa-penetrating EGC. In particular, patients with Pen A type early gastric cancer, which has been shown to be at high risk for lymph node metastases (LNM), should undergo D2 lymphadenectomy to achieve radicality.

Current methods for a pretreatment evaluation of LNM in EGC are insufficient due to suboptimal accuracy [29, 30], which may result in high rate of overtreatment and unnecessary gastrectomies.

Thus, the assessment of prognostic markers should be mandatory in the decision making, to better stratify EGCs and to give the patient the most adequate treatment.

In the present case series of EGC with a 10-year follow-up, neither LNM nor penetrating-subtype A were significantly associated with DFS, whereas patients with higher age and a tumor localization in the upper-middle part of the stomach present a higher risk of relapse or death from any cause. In the last 10 years, several models, based on clinico-radiological parameters, have been proposed as an attempt to predict the curability of the endoscopic treatment [31–33]. However, they did not consider in their analysis genomic or molecular data which could have helped in improving the prognostic accuracy of the model.

Several efforts have been made focusing on the biological characteristics which can impact the higher aggressiveness of some EGCs. Chen and collaborators showed the association between the collagen signature in the tumor microenvironment is an independent risk factor for LNM in EGC [12]. Moreover, it has been recently shown that LNM in EGC are related to a specific 3-markers DNA methylation signature, which can have a diagnostic potential to identify more aggressive EGC patients, thus reducing unnecessary gastrectomy in EGC [13].

Recent results revealed that a deep molecular characterization is useful in determining and predicting the malignant potential of EGC. Datta et al. examined the EGCs associated with the worst prognosis, comparing them with those with the best survival, with the aim of revealing a distinct genomic profile in those groups. The authors found that *TP53*^MUT/LOH^ was associated with those EGC with poor prognosis, in line with what we observed in our previous study, i.e., a prevalence of *TP53*^MUT/LOH^ type in the most aggressive EGC subtype, Pen A [14].

In the present study, we performed a wide NGS profiling to characterize the molecular landscape of submucosa-penetrating EGC to find if a specific genomic signature is related to a different prognosis.

Consistent with previous studies [34, 35], in our cohort of patients, *TP53* was the most frequently altered gene (52%), followed by other tumor suppressor mutated genes, such as *FBW7*, *RNF43* and *ARID1A*, already identified as biomarkers for early GC carcinogenesis, determinants for the progression of intestinal metaplasia to gastric cancer and worst prognosis [36–38]. The approach we adopted in the present study permitted us to evaluate the *TP53* entire coding region, but not its LOH. No significant association was observed between *TP53* mutations and relapse or Pen subtype.

In line with the enrichment of chromosomal unstable subtypes in *TP53* mutated tumors [39], we also observed frequent *ERBB2* and *CCN1E* focal amplifications in this case series, albeit without any association with prognosis.

Among the remaining genes, mutations we found in *LRP1B* seem especially of interest. *LRP1B* is a novel candidate tumor suppressor gene, capable of inhibiting tumor cell invasion and metastasis. Its reduced expression and alterations were more commonly found in tumors with a high lymph node ratio [36]. Moreover, *LRP1B* mutations have been found to be associated with an older age and a low survival rate [40]. This is roughly in line with our observation that *LRP1B* alterations tend to exhibit higher hazard of relapse or death from any cause even though the statistical tests did not reach significance level (*p* = 0.089). Being one of the most commonly mutated genes in our dataset overall, *LRP1B* is mutated at a high rate in three out of four clusters identified in the t-SNE embedding (Supplementary Table S6). Therefore, a potentially increased risk of relapse or death due to LRP1B would likely not be discernible in direct comparison of the clusters. Cluster 4, which does not contain any patients with mutated *LRP1B*, does at first seemingly display higher DFS in the first 50 months of follow-up compared to other clusters (Supplementary Figure S1). However, this cluster consists of only five patients and consequently has very few events, and the difference is not statistically significant when considering the complete duration of the follow-up.

Another interesting marker we identified in the present study is *ARID1A*, which is a member of the SW/SNF family and a key component of the adenosine triphosphate-dependent chromatin-remodeling complex. Loss of ARID1A expression is one of most frequent abnormalities associated with histological heterogeneity in GC, with mutation prevalence ranging from 8 to 31% [41], specifically 22% in our case series. Inactivating mutations in the gene, as well as complete or partial loss of ARID1A expression, seem to be an important factor promoting lymph node metastasis after invasion and are associated with poor patient prognosis [42]. Some authors indicate ARID1A’s role as a prognostic biomarker for the identification of high-risk GC patients, especially in early-stage undifferentiated cases [43]. Others found that somatic genomic alterations of *ARID1A* and other genes, combined with TMB, were associated with the development of metachronous cancers after curative endoscopic submucosal dissection and successful HP eradication in EGC [44]. It has been demonstrated that *ARID1A* alterations compromise the mismatch repair system; thus *ARID1A* defects are associated with MSI and high TMB, which can ultimately affect disease-free survival and overall survival of patients with advanced gastric cancer [45]. Interestingly, in our case series of EGC, we observed that *ARID1A* is mutated only in the most aggressive subtype Pen A. Furthermore, even the statistical significance was not reached, Pen A tumors more frequently present higher TMB values, higher frequency of MSI-subtypes and an overall increase in PI scores. Even if the results of pathways analysis should be interpreted with caution, the grouping of individual pathways according to their Top Level pathway seemingly identifies, among the others, a significant difference in the potential disruption to “Immune System” pathways in Pen A patients compared to Pen B patients. This is in line with the hypothesis that the loss of ARID1A leads to immune resistance, potentially because of the AKT signaling pathway activation, which could be finally related to the observed poor prognosis of ARID1A-negative patients [46]. The link between the loss of ARID1A and MSI and TMB, together with PD-L1 expression, TILs and systemic inflammatory markers [46–48] increases its clinical and prognostic relevance as a marker for screening and therapeutic response to targeted therapy and immune checkpoint inhibitors in gastric cancer.

To the best of our knowledge, our study is the largest to investigate the genomic alteration profile, including TMB and MSI status, in EGC patients with a relatively long follow-up. We are aware that the sample size of our cohort was limited, which is why we included publicly available data to parts of our analysis. However, large cohort studies are warranted to confirm our findings and elucidate the mechanisms by which these mutations impact the prognosis of EGC, particularly submucosa-penetrating tumors, with the ultimate goal of finding specific markers, easily determinable also in small diagnostic biopsy samples, which permit to offer the patient the best treatment option.

## Conclusions

In summary, the present study revealed the genomic characteristics of a small cohort of Italian EGC patients and suggested that *ARID1A* might warrant further investigations to provide a rationale for its use as a prognostic marker in EGC patients.

## Supplementary Information

Below is the link to the electronic supplementary material.

Supplementary file1 (DOCX 306 KB)

## Data Availability

Scripts used for the pathways instability analysis and Supplementary Tables 3 and 5 have been deposited in the following public depository 10.5281/zenodo.10816958.
