# Supplementary material for: Genomic events stratifying prognosis of early gastric cancer
Source: Gastric Cancer. 2024 Jul 19;27(6):1189–200. doi: 10.1007/s10120-024-01536-z (PMC11513700; doi:10.1007/s10120-024-01536-z)
Supplement: Supplementary file 1 — Supplementary file1 (DOCX 306 KB) [file 10120_2024_1536_MOESM1_ESM.docx]

**Molinari et al, Electronic Supplementary Material**

| **Supplementary Table S1.** TruSight Oncology 500 (TSO500) DNA panel gene list | | | | | | | | | | | | |
| --- | --- | --- | --- | --- | --- | --- | --- | --- | --- | --- | --- | --- |
| *ABL1* | *BCR* | ***CHEK1*** | *EPHA7* | ***FGF4*** | *GSK3B* | *IL7R* | *MAP3K1* | *NF2* | ***PIK3CA*** | *RAD51D* | *SMAD4* | *TGFBR2* |
| *ABL2* | *BIRC3* | ***CHEK2*** | *EPHB1* | ***FGF5*** | *H3F3A* | *INHA* | *MAP3K13* | *NFE2L2* | ***PIK3CB*** | *RAD52* | *SMARCA4* | *TMEM127* |
| *ACVR1* | *BLM* | *CIC* | ***ERBB2*** | ***FGF6*** | *H3F3B* | *INHBA* | *MAP3K14* | *NFKBIA* | *PIK3CD* | *RAD54L* | *SMARCB1* | *TMPRSS2* |
| *ACVR1B* | *BMPR1A* | *CREBBP* | ***ERBB3*** | ***FGF7*** | *H3F3C* | *INPP4A* | *MAP3K4* | *NKX2-1* | *PIK3CG* | ***RAF1*** | *SMARCD1* | *TNFAIP3* |
| *AKT1* | ***BRAF*** | *CRKL* | *ERBB4* | ***FGF8*** | *HGF* | *INPP4B* | *MAPK1* | *NKX3-1* | *PIK3R1* | *RANBP2* | *SMC1A* | *TNFRSF14* |
| ***AKT2*** | ***BRCA1*** | *CRLF2* | ***ERCC1*** | ***FGF9*** | *HIST1H1C* | *INSR* | *MAPK3* | *NOTCH1* | *PIK3R2* | *RARA* | *SMC3* | *TOP1* |
| *AKT3* | ***BRCA2*** | *CSF1R* | ***ERCC2*** | ***FGFR1*** | *HIST1H2BD* | *IRF2* | *MAX* | *NOTCH2* | *PIK3R3* | *RASA1* | *SMO* | *TOP2A* |
| ***ALK*** | *BRD4* | *CSF3R* | *ERCC3* | ***FGFR2*** | *HIST1H3A* | *IRF4* | *MCL1* | *NOTCH3* | *PIM1* | *RB1* | *SNCAIP* | *TP53* |
| *ALOX12B* | *BRIP1* | *CSNK1A1* | *ERCC4* | ***FGFR3*** | *HIST1H3B* | *IRS1* | *MDC1* | *NOTCH4* | *PLCG2* | *RBM10* | *SOCS1* | *TP63* |
| *ANKRD11* | *BTG1* | *CTCF* | *ERCC5* | ***FGFR4*** | *HIST1H3C* | *IRS2* | ***MDM2*** | *NPM1* | *PLK2* | *RECQL4* | *SOX10* | *TRAF2* |
| *ANKRD26* | *BTK* | *CTLA4* | *ERG* | *FH* | *HIST1H3D* | *JAK1* | ***MDM4*** | ***NRAS*** | *PMAIP1* | *REL* | *SOX17* | *TRAF7* |
| *APC* | *C11orf30* | *CTNNA1* | *ERRFI1* | *FLCN* | *HIST1H3E* | ***JAK2*** | *MED12* | ***NRG1*** | *PMS1* | ***RET*** | *SOX2* | *TSC1* |
| ***AR*** | *CALR* | *CTNNB1* | ***ESR1*** | *FLI1* | *HIST1H3F* | *JAK3* | *MEF2B* | *NSD1* | *PMS2* | *RFWD2* | *SOX9* | *TSC2* |
| *ARAF* | *CARD11* | *CUL3* | *ETS1* | *FLT1* | *HIST1H3G* | *JUN* | *MEN1* | *NTRK1* | *PNRC1* | *RHEB* | *SPEN* | *TSHR* |
| *ARFRP1* | *CASP8* | *CUX1* | *ETV1* | *FLT3* | *HIST1H3H* | *KAT6A* | ***MET*** | *NTRK2* | *POLD1* | *RHOA* | *SPOP* | *U2AF1* |
| *ARID1A* | *CBFB* | *CXCR4* | *ETV4* | *FLT4* | *HIST1H3I* | *KDM5A* | *MGA* | *NTRK3* | *POLE* | ***RICTOR*** | *SPTA1* | *VEGFA* |
| *ARID1B* | *CBL* | *CYLD* | *ETV5* | *FOXA1* | *HIST1H3J* | *KDM5C* | *MITF* | *NUP93* | *PPARG* | *RIT1* | *SRC* | *VHL* |
| *ARID2* | ***CCND1*** | *DAXX* | *ETV6* | *FOXL2* | *HIST2H3A* | *KDM6A* | *MLH1* | *NUTM1* | *PPM1D* | *RNF43* | *SRSF2* | *VTCN1* |
| *ARID5B* | *CCND2* | *DCUN1D1* | *EWSR1* | *FOXO1* | *HIST2H3C* | *KDR* | *MLL* | *PAK1* | *PPP2R1A* | *ROS1* | *STAG1* | *WISP3* |
| *ASXL1* | ***CCND3*** | *DDR2* | *EZH2* | *FOXP1* | *HIST2H3D* | *KEAP1* | *MLLT3* | *PAK3* | *PPP2R2A* | *RPS6KA4* | *STAG2* | *WT1* |
| *ASXL2* | ***CCNE1*** | *DDX41* | *FAM123B* | *FRS2* | *HIST3H3* | *KEL* | *MPL* | *PAK7* | *PPP6C* | ***RPS6KB1*** | *STAT3* | *XIAP* |
| ***ATM*** | *CD274* | *DHX15* | *FAM175A* | *FUBP1* | *HLA-A* | *KIF5B* | *MRE11A* | *PALB2* | *PRDM1* | *RPS6KB2* | *STAT4* | *XPO1* |
| *ATR* | *CD276* | *DICER1* | *FAM46C* | *FYN* | *HLA-B* | ***KIT*** | *MSH2* | *PARK2* | *PREX2* | *RPTOR* | *STAT5A* | *XRCC2* |
| *ATRX* | *CD74* | *DIS3* | *FANCA* | *GABRA6* | *HLA-C* | *KLF4* | *MSH3* | *PARP1* | *PRKAR1A* | *RUNX1* | *STAT5B* | *YAP1* |
| *AURKA* | *CD79A* | *DNAJB1* | *FANCC* | *GATA1* | *HNF1A* | *KLHL6* | *MSH6* | *PAX3* | *PRKCI* | *RUNX1T1* | *STK11* | *YES1* |
| *AURKB* | *CD79B* | *DNMT1* | *FANCD2* | *GATA2* | *HNRNPK* | *KMT2B* | *MST1* | *PAX5* | *PRKDC* | *RYBP* | *STK40* | *ZBTB2* |
| *AXIN1* | *CDC73* | *DNMT3A* | *FANCE* | *GATA3* | *HOXB13* | *KMT2C* | *MST1R* | *PAX7* | *PRSS8* | *SDHA* | *SUFU* | *ZBTB7A* |
| *AXIN2* | *CDH1* | *DNMT3B* | *FANCF* | *GATA4* | *HRAS* | *KMT2D* | *MTOR* | *PAX8* | *PTCH1* | *SDHAF2* | *SUZ12* | *ZFHX3* |
| *AXL* | *CDK12* | *DOT1L* | *FANCG* | *GATA6* | *HSD3B1* | ***KRAS*** | *MUTYH* | *PBRM1* | ***PTEN*** | *SDHB* | *SYK* | *ZNF217* |
| *B2M* | ***CDK4*** | *E2F3* | *FANCI* | *GEN1* | *HSP90AA1* | ***LAMP1*** | *MYB* | *PDCD1* | *PTPN11* | *SDHC* | *TAF1* | *ZNF703* |
| *BAP1* | ***CDK6*** | *EED* | *FANCL* | *GID4* | *ICOSLG* | *LATS1* | ***MYC*** | *PDCD1LG2* | *PTPRD* | *SDHD* | *TBX3* | *ZRSR2* |
| *BARD1* | *CDK8* | *EGFL7* | *FAS* | *GLI1* | *ID3* | *LATS2* | ***MYCL1*** | ***PDGFRA*** | *PTPRS* | *SETBP1* | *TCEB1* |  |
| *BBC3* | *CDKN1A* | ***EGFR*** | *FAT1* | *GNA11* | *IDH1* | *LMO1* | ***MYCN*** | ***PDGFRB*** | *PTPRT* | *SETD2* | *TCF3* |  |
| *BCL10* | *CDKN1B* | *EIF1AX* | *FBXW7* | *GNA13* | *IDH2* | *LRP1B* | *MYD88* | *PDK1* | *QKI* | *SF3B1* | *TCF7L2* |  |
| *BCL2* | *CDKN2A* | *EIF4A2* | ***FGF1*** | *GNAQ* | *IFNGR1* | *LYN* | *MYOD1* | *PDPK1* | *RAB35* | *SH2B3* | *TERC* |  |
| *BCL2L1* | *CDKN2B* | *EIF4E* | ***FGF10*** | *GNAS* | *IGF1* | *LZTR1* | *NAB2* | *PGR* | *RAC1* | *SH2D1A* | *TERT* |  |
| *BCL2L11* | *CDKN2C* | *EML4* | ***FGF14*** | *GPR124* | *IGF1R* | *MAGI2* | *NBN* | *PHF6* | *RAD21* | *SHQ1* | *TET1* |  |
| *BCL2L2* | *CEBPA* | *EP300* | ***FGF19*** | *GPS2* | *IGF2* | *MALT1* | *NCOA3* | *PHOX2B* | *RAD50* | *SLIT2* | *TET2* |  |
| *BCL6* | *CENPA* | *EPCAM* | ***FGF2*** | *GREM1* | *IKBKE* | *MAP2K1* | *NCOR1* | *PIK3C2B* | *RAD51* | *SLX4* | *TFE3* |  |
| *BCOR* | *CHD2* | *EPHA3* | ***FGF23*** | *GRIN2A* | *IKZF1* | *MAP2K2* | *NEGR1* | *PIK3C2G* | *RAD51B* | *SMAD2* | ***TFRC*** |  |
| *BCORL1* | *CHD4* | *EPHA5* | ***FGF3*** | *GRM3* | *IL10* | *MAP2K4* | *NF1* | *PIK3C3* | *RAD51C* | *SMAD3* | *TGFBR1* |  |

| **Supplementary Table S2.** Data of genomic instability in the case series | | | | | | | | |
| --- | --- | --- | --- | --- | --- | --- | --- | --- |
| **Molecular features** | **Pen B**  (n=14) (%) | | **Pen A**  (n=13)(%) | | | **Total**  (n=27) (%) | | ***p-value*** |
| **TMB** |  |  | |  |  |  |  |  |
| Median [IQ-IIIQ] | 5.2 [2.4 - 11.1] | | | 8 [4.8 - 25.3] | | 5.7 [4 - 12.7] | | 0.120 |
| min-max | 0.8 - 79.1 | | | 0.8 - 110.5 | | 0.8 - 110.5 | |  |
| < 10 | 10 (71.4) | | | 8 (61.5) | | 18 (66.7) | | 0.695 |
| ≥ 10 | 4 (28.6) | | | 5 (38.5) | | 9 (33.3) | |  |
| **MSI** |  |  | |  |  |  |  |  |
| Median [IQ-IIIQ] | 2.6 [1.8 - 3.8] | | | 2.8 [1.8 - 22.3] | | 2.6 [1.8 - 4.8] | | 0.423 |
| min-max | 0 - 43.0 | | | 0.9 - 70.4 | | 0 - 70.4 | |  |
| ≤ 20% | 13 (92.9) | | | 9 (69.2) | | 22 (81.5) | | 0.165 |
| >  20% | 1 (7.1) | | | 4 (30.8) | | 5 (18.5) | |  |

**Supplementary Figure S1.** Kaplan-Meier survival analysis of disease-free survival for 4 patient clusters obtained from the pathway instability analysis, as well as for EGC patients grouped according to Kodama classification.


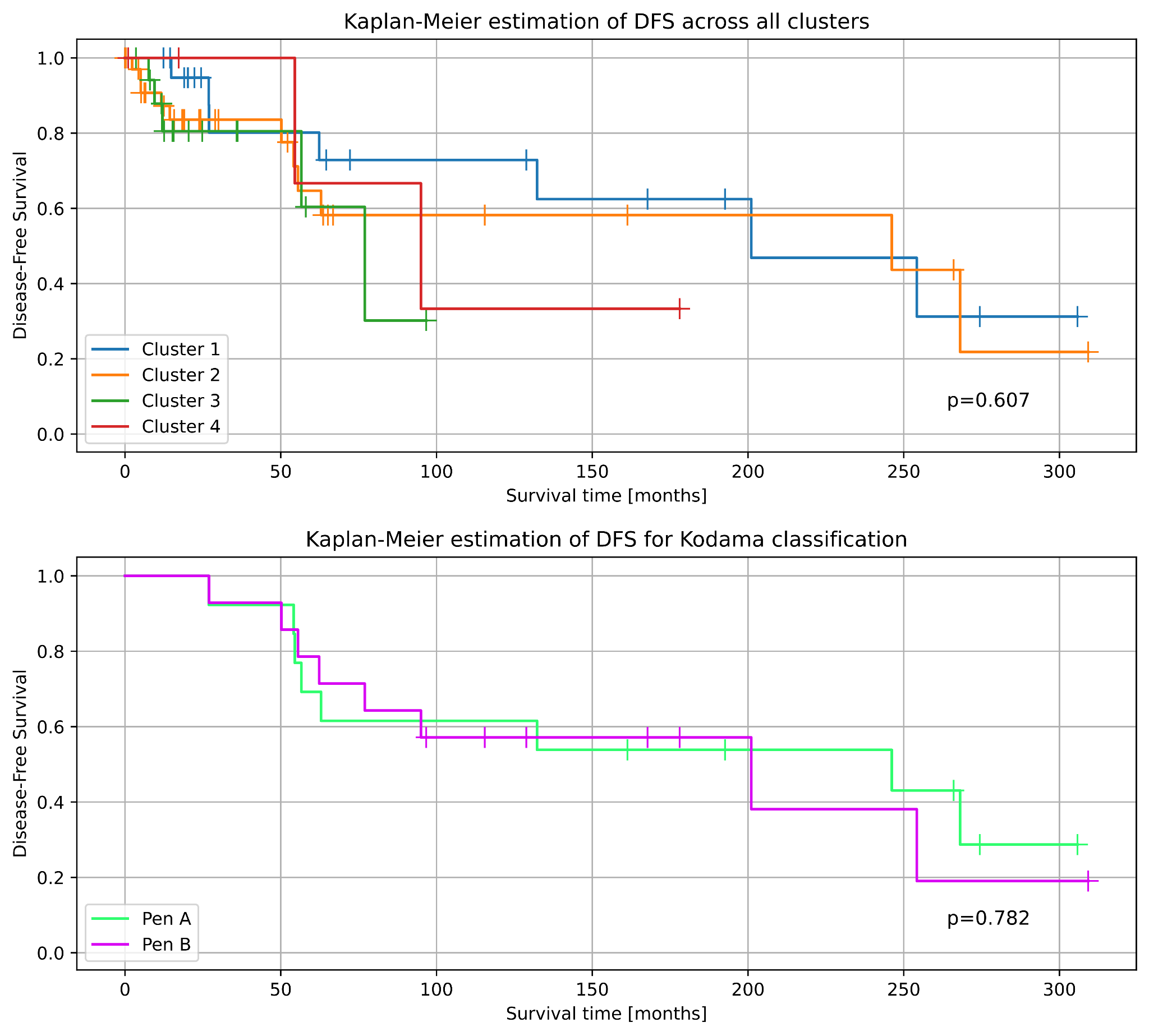


**Supplementary Table S3:** Results of the Wilcoxon rank-sum test comparing PI scores of individual pathways across TMB and MSI classes, Kodama classification and relapse status. Benjamini-Hochberg correction with FDR=0.05 was applied, but uncorrected p-values are also reported in the table. Furthermore, information on which genes from the TSO500 gene panel are present in each of the tested pathways is provided. Due to the large size of the table, it is made available in the Zenodo repository: [**https://zenodo.org/doi/10.5281/zenodo.10816958**](https://zenodo.org/doi/10.5281/zenodo.10816958)

**Supplementary Table S4.** Clinico-pathological characteristics of EGC from the present case series and  TCGA – PanCancer Atlas cohort. Upper-middle tumors correspond in cardia, fundus and body localization and a lower localization corresponds with an antrum tumor. Regarding pathological classification of TCGA tumors: Intestinal Type STAD, Tubular STAD and Papillary STAD are compared with “intestinal” EGC tumors; Diffuse Type STAD and Signet Ring Cell Carcinomas are associated with “diffuse” EGCs and STAD Not Specified are considerate as “other”.

| **Characteristics** | **EGC**  (n=27) (%) | **TCGA-EGC** (n=54) (%) | **Total** (n=81) (%) | ***p-value*** |
| --- | --- | --- | --- | --- |
| **Age at diagnosis (yrs)** |  |  |  |  |
| Mean +- S.D.^a^ | 67.0 +- 11.7 | 70.3 +- 10.5 | 69.2 +- 11.0 | 0.172 |
| Min-max | 41 – 87 | 45 – 90 | 41 – 90 |  |
|  |  |  |  |  |
| **Gender** |  |  |  |  |
| F | 15 (55.56) | 17 (31.48) | 32 (39.51) | 0.037 |
| M | 12 (44.44) | 37 (68.52) | 49 (60.49) |  |
|  |  |  |  |  |
| **Tumor location** |  |  |  |  |
| Upper-middle | 13 (48.15) | 38 (70.37) | 51 (62.96) | 0.015 |
| Lower | 14 (51.85) | 12 (22.22) | 26 (32.10) |  |
| Other | 0 (0) | 4 (7.41) | 4 (4.94) |  |
|  |  |  |  |  |
| **Lauren classification** |  |  |  |  |
| Intestinal | 23 (85.19) | 25 (46.30) | 48 (59.26) | 0.487 |
| Diffuse or mixed | 4 (14.81) | 7 (12.96) | 11 (13.58) |  |
| Other | 0 (0) | 22 (40.74) | 22 (27.16) |  |
|  |  |  |  |  |
| **Grade** |  |  |  |  |
| G1 + G2 | 14 (51.85) | 33 (61.11) | 47 (58.02) | 0.716 |
| G3 | 12 (44.44) | 19 (35.19) | 31 (38.27) |  |
| Unknown | 1 (3.70) | 2 (3.70) | 3 (3.70) |  |
|  |  |  |  |  |
| **pN status** |  |  |  |  |
| pN0 | 19 (70.37) | 51 (94.44) | 70 (86.42) | 0.003 |
| pN+ | 8 (29.63) | 3 (5.56) | 11 (13.58) |  |
|  |  |  |  |  |
| **TMB^b^ Status** |  |  |  |  |
| ≤ 10 | 18 (66.67) | 36 (66.67) | 54 (66.67) | 1 |
| > 10 | 9 (33.33) | 18 (33.33) | 27 (33.33) |  |
|  |  |  |  |  |
| **MSI^c^ Status** |  |  |  |  |
| < 20% | 22 (81.48) | 46 (85.19) | 68 (83.95) | 0.669 |
| ≥ 20% | 5 (18.52) | 8 (14.81) | 13 (16.05) |  |

^a^S.D.: standard deviation

^b^TMB: Tumor mutational burden

^c^MSI: microsatellite instability

**Supplementary Table S5:** Significantly disrupted pathways for each of the 4 clusters identified with t-SNE dimension reduction and DBSCAN clustering algorithm. The distribution of pathways between the clusters was performed using the Wilcoxon rank-sum test with Benjamini-Hochberg correction (FDR=0.5), and the q-values reported. Furthermore, the corresponding Reactome Top Level pathway for each significant pathway is indicated. The table is available in the Zenodo repository: [**https://zenodo.org/doi/10.5281/zenodo.1081695**](https://zenodo.org/doi/10.5281/zenodo.10816958)

**Supplementary Table S6.** Top 10 most commonly altered genes in the combined cohort of EGC and eGC-TCGA patients (n=81) for each of the 4 clusters identified by the pathway instability analysis and overall. Relative mutation frequency, indicating the percentage of patients within the cluster with at least 1 non-benign variation for a given gene, is given in parentheses. Mutation frequencies of multiple characteristic genes (TP53, ARID1A, RHOA) vary between the clusters, demonstrating different mutational landscapes.

|  | **Cluster 1 (%)** | **Cluster 2 (%)** | **Cluster 3 (%)** | **Cluster 4 (%)** | **Overall Distribution (%)** |
| --- | --- | --- | --- | --- | --- |
| 1. | TP53 (100.0) | ARID1A (44.44) | TP53 (100.0) | RHOA (100.0) | TP53 (50.62) |
| 2. | SPTA1 (22.73) | LRP1B (30.56) | LRP1B (33.33) | TP53 (20.0) | ARID1A (29.63) |
| 3. | LRP1B (18.18) | KMT2D (27.78) | EPHA5 (33.33) | RNF43 (20.0) | LRP1B (25.93) |
| 4. | ARID1A (18.18) | KMT2C (25.0) | RNF43 (27.78) | FBXW7 (20.0) | SPTA1 (18.52) |
| 5. | APC (13.64) | SPTA1 (22.22) | AR (27.78) | KMT2B (20.0) | KMT2C (17.28) |
| 6. | CARD11 (13.64) | FLT4 (22.22) | GRIN2A (27.78) | SPEN (20.0) | RNF43 (17.28) |
| 7. | HGF (13.64) | RNF43 (22.22) | FBXW7 (27.78) | NBN (20.0) | PTPRT (17.28) |
| 8. | PIK3CD (9.09) | HLA-B (19.44) | APC (22.22) | FAT1 (20.0) | PTPRS (17.28) |
| 9. | GATA3 (9.09) | ERBB3 (19.44) | PTPRT (22.22) | BRCA2 (20.0) | APC (16.05) |
| 10. | PTPRD (9.09) | PTPRT (19.44) | JAK1 (22.22) | RAD50 (20.0) | KMT2D (14.81) |

**Pathway Instability Analysis**

The PI score for a given pathway (*p*) is calculated by summing the normalized mutation rates (*nMR_n_*) of member genes (*n*) of the said pathway and dividing this sum by the number of member genes in a pathway (*N_p_*). Normalized mutation rate for a gene is just the count of detected non-benign variants of that gene  in a sample divided by the gene's CDS length in kbps which we obtain from Ensembl/Biomart (https://www.ensembl.org/biomart/martview/). In our implementation, we multiply the normalized mutation rates of genes with their raw CNV value (*CNV_n_*) if it is larger than 1, otherwise with its inverse:


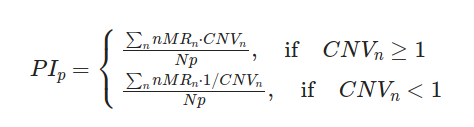


The original implementation of the PI score by *Zolotovskaia et al* does not consider CNV data. However, we propose that including CNV information presents an improvement to the PI score, as deviations from the normal copy number of the pathway's constituent genes contribute to increased disruption (i.e. instability) of the pathway.

Due to the hierarchical structure of the Reactome pathway database, individual pathways are grouped to one or more Top Level Pathways which represent a broad category of biological functions or processes. Using *rbioapi 0.8* **[Rezwani et al., doi: 10.1093/bioinformatics/btac172]**, corresponding Top Level Pathways were assigned to each of the 218 pathways identified by the enrichment analysis. Using the Wilcoxon rank-sum test, PI-score distribution of pathways grouped according to their Top Level Pathways was compared between relapsed and non-relapsed patients (TCGA-EGC dataset), and Pen A and Pen B patients (EGC dataset). For the case of TCGA-EGC patients, only those genes which are available in the TSO500 gene panel were used to calculate the PI scores.

**References**

Zolotovskaia, Marianna A., Maxim I. Sorokin, Sergey A. Roumiantsev, Nikolay M. Borisov, and Anton A. Buzdin. “Pathway Instability Is an Effective New Mutation-Based Type of Cancer Biomarkers.” Frontiers in Oncology 8 (January 4, 2019): 658.<https://doi.org/10.3389/fonc.2018.00658>.

Rezwani et al.*,* **doi: 10.1093/bioinformatics/btac172**
